# Campylobacteriosis Outbreak Associated with Consuming Undercooked Chicken Liver Pâté — Ohio and Oregon, December 2013–January 2014

**Published:** 2015-04-17

**Authors:** Magdalena Kendall Scott, Aimee Geissler, Tasha Poissant, Emilio DeBess, Beth Melius, Kaye Eckmann, Ellen Salehi, Paul R. Cieslak

**Affiliations:** 1 Oregon Public Health Division; 2 Division of Foodborne, Waterborne, and Environmental Diseases, National Center for Emerging and Zoonotic Infectious Diseases, CDC; 3 Washington State Department of Health; 4 Ohio Department of Health

On January 8, 2014, the Ohio Department of Health notified the Oregon Public Health Division (OPHD) of campylobacteriosis in two Ohio residents recently returned from Oregon. The travelers reported consuming chicken liver pâté* at an Oregon restaurant. On January 10, OPHD received additional reports of campylobacteriosis in two persons who had consumed chicken liver pâté at another Oregon restaurant. *Campylobacter jejuni* was isolated in cultures of fecal specimens from three patients. OPHD investigated to determine the sources of the illnesses and to institute preventive measures.

Both restaurants reported using undercooked chicken livers to prepare their pâté; an environmental health investigation revealed that the livers were purchased from the same U.S. Department of Agriculture Food Safety and Inspection Service (FSIS)–regulated establishment in the state of Washington. The establishment reported that livers were rinsed with a chlorine solution before packaging. However, culture of five of nine raw liver samples from both restaurants and from the establishment yielded *C. jejuni*; none of three pâté samples from the restaurants yielded *C. jejuni.* One human stool specimen and three liver samples were typed by pulsed-field gel electrophoresis (PFGE); the human isolate and one liver sample had indistinguishable PFGE patterns when digested by the restriction enzyme *Sma*I. The human isolate was susceptible to all antimicrobials tested by CDC’s National Antimicrobial Resistance Monitoring System.

A presumptive case was defined as diarrhea lasting >2 days, within 7 days after consumption of undercooked chicken liver; a confirmed case was defined as laboratory evidence of *C. jejuni* infection within 7 days after consumption of undercooked chicken liver. In all, three laboratory-confirmed and two presumptive cases of campylobacteriosis following consumption of chicken livers were reported in Ohio and Oregon. Illness onsets ranged from December 24, 2013, to January 17, 2014. Patient age range was 31–76 years; three were women. Based on OPHD’s recommendation, both restaurants voluntarily stopped serving liver. The FSIS-regulated establishment also voluntarily stopped selling chicken livers.

This is the second multistate outbreak of campylobacteriosis associated with consumption of undercooked chicken liver reported in the United States (1). Outbreaks caused by chicken liver pâté are well documented in Europe (2,3). Chicken livers and pâté should be considered inherently risky foods, given the methods by which they are routinely prepared. Pâté made with chicken liver is often undercooked to preserve texture. Consumers might be unable to discern whether pâté is cooked thoroughly because partially cooked livers might be blended with other ingredients and chilled. At FSIS-regulated establishments, such as the one involved in this outbreak, livers are inspected to ensure that they are free from visible signs of disease, but they are not required to be free from bacteria (4). A recent study isolated *Campylobacter* from 77% of chicken livers cultured (5). Washing is insufficient to render chicken livers safe for consumption; they should be cooked to an internal temperature of 165°F (74°C).

During the outbreak investigation, OPHD learned of a campylobacteriosis case in a Washington state resident who had eaten raw chicken livers that had been chopped into pill-sized pieces and frozen, as prescribed by a naturopathic physician. The livers were from the same establishment that supplied the Oregon restaurants. No isolate from the case was available for subtyping, but culture of frozen pieces of liver collected from this patient yielded *C. jejuni*.

This report illustrates that follow-up of possible outbreaks identified by routine interviewing by health departments can identify sources of illnesses and result in control measures that protect public health. *Campylobacter* is thought to be the most common bacterial cause of diarrheal illness in the United States (6), and infection is now nationally notifiable.

## Footnotes

* A spreadable paste made from cooked ground meat (often poultry livers) combined with various other ingredients.
